# Anti-Aging Effect and Mechanism of Proanthocyanidins Extracted from *Sea buckthorn* on Hydrogen Peroxide-Induced Aging Human Skin Fibroblasts

**DOI:** 10.3390/antiox11101900

**Published:** 2022-09-25

**Authors:** Xinying Liu, Yi Xing, Michael Yuen, Tina Yuen, Hywel Yuen, Qiang Peng

**Affiliations:** 1 College of Food Science and Engineering, Northwest A&F University, Yangling, Xianyang 712100, China; 2 Puredia Limited, Xining 810000, China

**Keywords:** *Sea buckthorn* proanthocyanidins, anti-aging, type I collagen, oxidative stress, reactive oxygen species

## Abstract

Oxidative stress is the leading cause of skin aging damage. Excessive accumulation of reactive oxygen species (ROS) in cells induced by hydrogen peroxide (H_2_O_2_) triggers a decrease in collagen synthesis and an increase in collagen degradation, which are biomarkers of skin aging. We evaluated the potential protective mechanism of *Sea buckthorn* proanthocyanidins (SBP) against the oxidative stress-induced skin aging process from multiple aspects. We treated human skin fibroblasts (HSFs) with 300 µmoL/L of H_2_O_2_ for 24 h, followed by 25, 50, and 100 µg/mL of SBP for 24 h. The results showed that SBP could enhance the activities of superoxide dismutase (SOD) and glutathione (GSH), effectively remove excess ROS, and significantly improve the changes in cell morphology and viability caused by excessive ROS in skin cells. In addition, SBP could promote the synthesis of Col I in aging HSFs through the TGF-β1/Smads pathway and inhibit the degradation of Col I by regulating the MMPs/TIMPs system, thereby maintaining the stability of the ECM structure to achieve anti-aging purposes. Finally, we studied the migration ability of SBP, and the results showed that 100 µg/mL of SBP was most conducive to the cell migration of senescent cells, laying a foundation for follow-up animal experiments. These results will increase the application value of SBP in the cosmetic and antioxidative functional food industries.

## 1. Introduction

Skin aging is a skin problem that is gaining increasing concern. Two reasons cause the process of skin aging: one is natural aging that occurs over time and the other is extrinsic aging, a process that occurs due to environmental conditions, such as prolonged sunlight exposure and smoking, which increase reactive oxygen species (ROS) in cells, resulting in oxidative stress. This affects the contents of antioxidants and oxidative enzymes in cells, such as glutathione (GSH) and superoxide substance dismutase (SOD), which will cause damage to cells, thereby accelerating cell aging [1]. Hydrogen peroxide (H_2_O_2)_, as a ROS that readily induces free radical production and lipid peroxidation through cell membranes, is often used to induce oxidative stress and cellular senescence in in vitro models [2]. Fibroblasts are the main cells in the dermis that can synthesize collagen (percentage of type I collagen (Col I) can reach up to 90%), which delays cell aging and maintains and provides skin elasticity [3]. Therefore, assessing collagen changes in cells, either reduced synthesis or increased degradation, is a critical factor to analyze. The TGF-β/Smads pathway is a major regulator in type I procollagen synthesis in human skin. TGF-β initiates cellular action by binding to specific cell surface receptor complexes, thereby phosphorylating the transcription factor Smad3. Phosphorylated Smad3 (p-Smad3) binds to Smad4, thereby affecting the transcription and expression of type I procollagen, which in turn affects the content of Col I [4].

On the other hand, related studies have found that normal collagen degradation is led by matrix metalloproteinases (MMP), a family of zinc-containing proteases that specifically degrade extracellular matrix proteins, including connective tissue [5]. An increased ROS content in cells stimulates the overexpression of MMP, and the degradation of type I collagen is initiated by MMP-1 and continued by MMP-3 [6]. Furthermore, the effects of MMP-1 and MMP-3 are inhibited by tissue inhibitor of metalloproteinase-1 (TIMP-1) [7]. Therefore, studying the synthesis and degradation of Col I may serve as a new method for preventing and treating skin aging.

*Sea buckthorn* (Hippophae, Elaeagnaceae) is widely cultivated in Europe, Canada, and the United States. It is popular in the food and pharmaceutical industries due to its richness in various bioactive components, such as vitamins, amino acids, and flavonoids [8]. High concentrations of proanthocyanidins are found in *Sea buckthorn* seeds, roots, flowers, green berries, and stems [9]. In recent years, related studies discovered that proanthocyanidins have a variety of physiological functions and can thus be used to treat cancer and cardiovascular diseases [10], protect retinal cells from photodamage [11], prevent or treat diabetic nephropathy [12], promote weight loss through modulating adipose thermogenesis and gut microbiota [13], as well as inhibit capillary hyperpermeability, lipid peroxidation, and platelet aggregation [14]. A few studies are focusing on the use of SBP in delaying skin aging. Zhu [15] from our team found that SBP provides significant protection against oxidative stress in RAW264.7 cells caused by an excessive ROS content in the cells via reacting with excessive H_2_O_2_, but the mechanism is still unclear. Therefore, we established an aging model by treating human skin fibroblasts (HSFs) with different concentrations of H_2_O_2_ to study the protective effect and mechanism of SBP on the aging of HSFs induced by H_2_O_2_ and to investigate its role in preventing cell aging. This paper will discuss possible SBP applications and their market value in the cosmetic industry and healthcare products industry.

## 2. Materials and Methods

### 2.1. Materials

*Sea Buckthorn* proanthocyanidins (SBP, Purity: 91.5%) were provided by Puredia Limited (Qinghai, China). The SBP was extracted from *Sea buckthorn* by water extraction and macroporous resin column chromatography (MRCC), trademarked as CyanthOx™. HSFs (derived from human superficial skin tissue), fetal bovine serum (FBS), Dulbecco’s modified Eagle medium (DMEM), phosphate-buffered saline (PBS), penicillin–streptomycin solution (double antibody) 100×, and 0.25% trypsin solution were purchased from Xiamen Immocell Biotechnology Co., Ltd. (Xiamen, China). A Cell Counting Kit 8 (CCK-8) ROS detection kit was purchased from Beijing Solar Science & Technology Co., Ltd. (Beijing, China). A senescence β-galactosidase staining kit, SOD assay kit and WST-8, GSH assay kit, malondialdehyde (MDA) assay kit, and particular fixative solution, washing solution, blocking solution, Alexa Fluor 488-labeled Goat Anti-Rabbit IgG(H+L), and antifade mounting medium with DAPI for immunofluorescence staining were all purchased from Beyotime Inc. (Shanghai, China). Primary antibodies, including anti-TGF-β1, anti-Smad 3, anti-phospho-Smad 3 (Ser425), anti-Smad 4, anti-type I collagen, and β-actin, were obtained from Affinity Biosciences (OH, USA). MMP-1, MMP-3, and TIMP-1 Elisa assay kits were obtained from Elabscience Biotechnology Co., Ltd. (Wuhan, China).

### 2.2. Cell Culture

After resuscitation, HSFs were placed in a high-glucose DMEM medium supplemented with 10% PBS and 0.1% penicillin–streptomycin; then, they were incubated at a constant temperature of 37 °C with a 5% CO_2_ cell incubator. Cell passage or subsequent experiments were conducted when the cell confluency was greater than 80%.

### 2.3. Cell Viability Assay

A Cell Counting Kit 8 was used to measure the cell viability. The drugs were added to the cells, and after culturing for 24 h, 10 μL of CCK-8 reagent was added to each well. The culture plate was incubated in a cell incubator (37 °C with 5% CO_2_) for 2 h. To calculate the cell viability, the absorbance at 450 nm was measured with a microplate reader (PowerWave XS, Bio-Tek, Winooski, VT, USA).

### 2.4. Cytotoxicity Assay of SBP

HSFs in the logarithmic growth phase were seeded in 96-well plates (100 μL per well), and the cell concentration was 1.0 × 10^5^ cells/mL. The 96-well plate loaded with cells was placed in a carbon dioxide constant-temperature incubator at 37 °C with 5% CO_2_ for 24 h, followed by PBS washing twice. The supernatant was discarded before washing. An amount of 100 μL of the samples in different concentrations (50, 100, 200, 400, and 800 μg/mL) was added accordingly. The control group contained the culture medium only. The cell viability was then detected by a CCK-8 assay after culturing for 24 h.

### 2.5. Establishment of Senescent Cell Models

HSFs in the logarithmic growth phase were seeded in 96-well plates at 100 μL per well and the cell concentration was 1.0 × 10^5^ cells/mL. The cells were cultured at 37 °C under 5% CO_2_ for 24 h until the cells adhered, followed by PBS washing twice. The supernatant was discarded before washing. H_2_O_2_ solutions in different concentrations prepared in medium were added. The following 9 concentration gradient groups were set: 0 (blank), 100, 200, 300, 400, 500, 600, 700, and 800 µmol/L, and three replicate wells were set for each concentration gradient. After 24 h of cell cultivation, the cell viability was detected by a CCK-8 assay.

### 2.6. Cell Treatment

HSFs in the logarithmic growth phase were seeded in a 96-well plate at 100 μL per well and the cell concentration was 1.0 × 105 cells/mL. The cells were cultured at 37 °C under 5% CO_2_ for 24 h until the cells adhered, and the supernatant was discarded, washed twice with PBS, and then treated with drugs. A total of five experimental groups were divided: (1) control group, with only a culture medium added; (2) in the aging model group, 300 µmol/L of H_2_O_2_ was added to the culture medium; (3) 300 µmol/L of H_2_O_2_ and 25 µg/mL of SBP were added to the culture medium; (4) 300 μmol/L H_2_O_2_ and 50 μg/mL SBP were added to the culture medium; and (5) 300 µmol/L of H_2_O_2_ and 100 µg/mL of SBP were added to the culture medium.

### 2.7. Effects of SBP on H_2_O_2_-Induced Senescent HSFs

The five groups of cells were treated with drugs and cultured for 24 h. The cell viability was detected by the CCK-8 method, and the cell morphology of each group was observed under an inverted microscope.

### 2.8. β-Galactosidase Staining Method to Verify the Effect of SBP on H_2_O_2_-Induced Senescent HSFs

β-Galactosidase staining is a method for staining senescent cells or tissues based on the up-regulation of SA-β-Gal activity in senescent cells [16]. The specific experimental steps were carried out following the kit’s instructions. The cells were fixed in the fixative solution for 15 min, washed with PBS, and then 1 mL of freshly prepared SA-β-gal staining solution (containing 10 µL β-galactosidase-stained solutions A and B, 930 µL solution C, and 50 µL X-gal solution) was added. The cells were then rinsed with PBS for 10 min after being protected from light overnight at 37 °C. An inverted light microscope was then used to observe the treated cells. 

### 2.9. ROS Assay

The five groups of cells were treated with drugs and then harvested after 24 h of culturing. First, the supernatant was discarded from the cell culture medium. Then, after washing twice with PBS, culture medium (serum-free) containing DCFH-DA (10 μM) was added to each well. The cells were then observed with a green fluorescence microscope (observed via fluorescence microscopy IX71-F22FL/PH, Olympus, Tokyo, Japan). After the cells in each well were pipetted, an F-7000 fluorescence spectrophotometer was used for detection (emission wavelength: 530 nm; excitation wavelength: 485 nm). 

### 2.10. Determination of SOD Activity, GSH, and MDA Content

Drugs were added to the five groups of cells and they were then cultured for 24 h. After incubation, the supernatant was discarded and 200 µL of cell lysate was added to each well. Then, the lysate was collected and centrifuged at 12,000 rpm and 4 °C for 10 min, and the supernatant was collected as a sample for testing. Specific experimental steps were followed to determine the SOD activity, GSH, and MDA content following the kit’s instructions.

### 2.11. Col I Immunofluorescence Staining

The five groups of cells were treated with drugs for 24 h, followed by immunofluorescence experiments. First, the old culture medium was aspirated and discarded. Next, the cells were washed three times with PBS and fixed with a special fixative for immunofluorescence for 10 min; after removing the fixative, they were washed three times with a washing solution for 3–5 min each time. Next, a blocking buffer was added (blocking time: 60 min), followed by the primary antibody Col I antibody (1:200) for another 60 min. After removing the primary antibody, the cells were washed, 1 mL of fluorescently labeled secondary antibody was added (Alexa Fluor 488-labeled Goat Anti-Rabbit IgG(H+L), 1:200), and the cells were incubated at 37 °C for 60 min in the dark. Finally, the cells’ nuclei were stained and blocked with an anti-fluorescence quenching agent (DAPI containing). The result was observed and photographed with an inverted fluorescence microscope. The mean fluorescent intensity of each group was expressed with the help of ImageJ software (Rawak Software, Inc., Stuttgart, Germany). 

### 2.12. Measurement of MMP-1, -3, and TIMP-1 Production

An enzyme-linked immunosorbent assay (ELISA) was used to detect the amounts of MMP-1, MMP-3, and TIMP-1 in each group of cells. The specific experimental steps were carried out following the kit’s instructions.

### 2.13. Western Blotting

According to Menicacci, B. et al. [17], five groups of cells were treated with drugs, and the cells were harvested after 24 h of culturing. Radioimmunoprecipitation assay buffer (RIPA buffer) (Bioworld technology, St. Louis Park, MN, USA)-induced lysis was performed for 30 min, and the supernatant was collected after centrifugation at 12,000 rpm for 5 min. The protein content of the lysates was measured using a BCA protein assay kit (Biotopped, Beijing, China) following the manufacturer’s protocol. Equal amounts of protein standard solutions were loaded into the corresponding lanes, run under 10% SDS polyacrylamide gel electrophoresis, then transferred to polyvinylidene fluoride membranes and blocked for one hour at room temperature (25–36 °C). The membranes were then mixed with specific primary antibodies—anti-TGF-β1 (1:2000), anti-Smad3 (1:2000), anti-phospho-Smad3 (Ser425) (1:2000), anti-Smad4 (1:2000), Type I collagen (1:1000), and anti-β-actin (1:5000) antibodies—overnight at 4 °C. The membrane was then incubated with secondary antibody HRP-conjugated Goat Anti-Rabbit IgG(H+L) (1:5000, Proteintech Group, Wuhan, China) for one hour at room temperature. Protein bands were visualized using an ECL Western Blot Detection Kit (1:5000, Gsebio, Xi’an, Shanxi, China).

### 2.14. Cell Wound Scratch Assay

Scratch experiments were conducted to analyze the migration ability of SBP-treated HSFs. Simplifying the method from Pan et al. [18], the HSFs in the logarithmic growth phase were seeded into 6-well plates at a concentration of 1.0×10^5^ cells/well. After 24 h of culturing, cell scratches were created with a 10 µL pipette tip perpendicular to the plate, and the width of each scratch was as consistent as possible. Then, each well was washed twice with PBS to remove loose cell debris. Finally, a serum-free medium containing 300 µmol/L H_2_O_2_ or 100 µg/mL SBP drug was added. After 24 h, cell migration was observed using an inverted microscope (magnification, ×20), and the scratch area was analyzed using ImageJ software. We calculated the cell migration rate as follows:

(1) $$\mathrm{Cell}\;\mathrm{migration}\;\mathrm{rate}=\frac{0\;h\;\mathrm{scratch}\;\mathrm{width}-\mathrm{scratch}\;\mathrm{width}\;\mathrm{after}\;\mathrm{culture}}{0\;h\;\mathrm{scratch}\;\mathrm{width}\;}\times 100\%$$

### 2.15. Statistical Analysis

All data were entered into and analyzed using SPSS 22.0 software (SPSS Inc., Chicago, IL, United States). After the data had been tested for normal distributions, one-way analysis of variance (ANOVA) and Tukey post hoc tests were used to compare the differences between the overall groups. All trials were conducted in triplicate, and the statistical means and standard deviations were calculated.

## 3. Results

### 3.1. Cytotoxicity Assay of SBP on HSFs and Establishment of Senescent Cell Models

**Figure 1:**
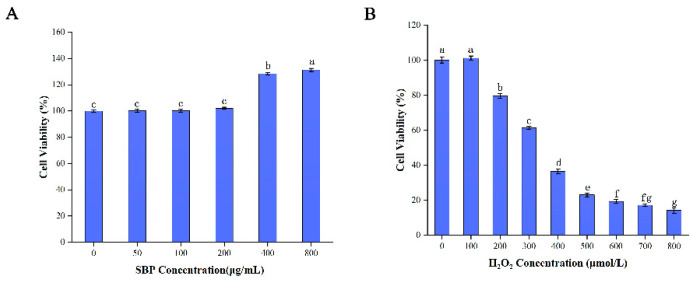
Toxic effects of SBP on HSFs and the establishment of an aging model. (**A**) The toxic effects of different concentrations of SBP on HSFs were detected by the CCK-8 method; (**B**) the effect of different concentrations of H_2_O_2_ on the cell viability of HSFs was detected by the CCK-8 method. Results are the mean ± SD of three independent experiments. Bars with different letters are significantly different (*p* < 0.05).

To examine the toxic effects of SBP on HSFs, we set the non-toxic concentration range to be 80% of the HSFs being alive after different experimental treatments. We selected the concentration range of SBP from 0 to 800 µg/mL to detect the cell viability of HSFs using the CCK-8 method. The results are shown in Figure 1A, and indicate that SBP can promote the proliferation of HSFs at concentrations of 400 and 800 μg/mL, and the cell viability reached about 128% and 131%, respectively, indicating that SBP may favor the growth of skin cells. When the SBP concentration reached 50 μg/mL and 100 μg/mL, the effect on the viability of HSFs cells was not significantly different from that of the blank control group. Therefore, we speculated that the SBP concentration of 25 μg/mL was consistent with the effects of the 50 μg/mL and 100 μg/mL concentrations on cell viability. To achieve cell non-toxicity and ensure cell number consistency in subsequent experiments, we selected 25, 50, and 100 μg/mL SBP as the concentrations for subsequent studies.

To verify the positive effect of SBP on senescent cells, we treated HSFs with H_2_O_2_ in the concentration range of 0 to 800 µmol/L to establish a H_2_O_2_-induced senescence model and detected their cell viability using the CCK-8 assay. As shown in Figure 1B, the cell viability decreased gradually with the increase in the H_2_O_2_ concentration from 100 μmol/L to 600 μmol/L. For example, when the H_2_O_2_ concentration reached 300 µmoL/L, the cell viability reached 61.45%. When the H_2_O_2_ concentration reached 400 µmoL/L, the cell viability decreased to 36.58%. Therefore, the half inhibitory concentration of H_2_O_2_ should lie between 300 and 400 µmoL/L. For the convenience of subsequent experiments, we selected 300 μmol/L H_2_O_2_ to establish a H_2_O_2_-induced senescence model.

### 3.2. SBP Favors the Restoration of Cell Morphology and Cell Viability in H_2_O_2_-Induced Senescent Cells

**Figure 2:**
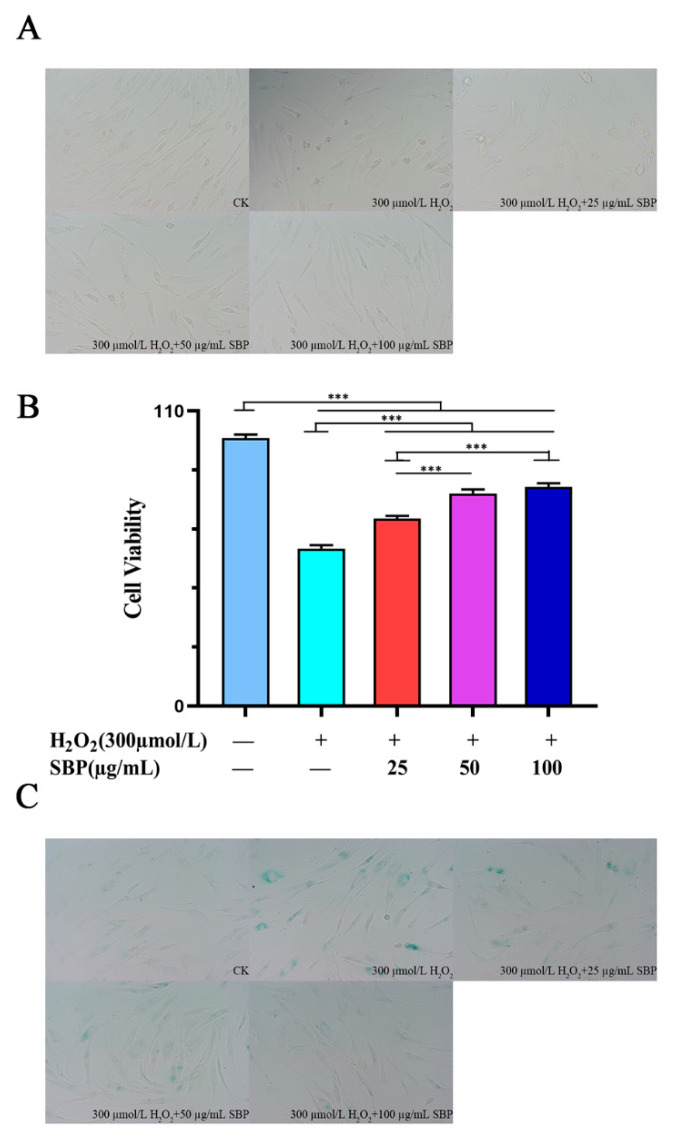
Cell morphology and cell viability of SBP in H_2_O_2_-induced senescent cells. (**A**) Effects of different SBP concentrations on cell morphology in H_2_O_2_-induced senescence HSFs observed by an ordinary light microscope. (**B**) CCK-8 method to detect the effects of different SBP concentrations on cell viability in H_2_O_2_-induced senescent HSFs. (**C**) β-galactosidase staining method to verify the anti-aging effect of different SBP concentrations on H_2_O_2_-induced senescent HSFs. In the figure “+” means added, “−” means not added. The results are the mean ± SD of three independent experiments. *** *p* < 0.001 indicate significant differences compared with the various experimental groups.

Cellular senescence alters cell morphology. As shown in Figure 2A,C, cells in the blank control group had large cell bodies. They were spindle- or star-shaped flat cells with multiple protrusions. In addition, regular oval nuclei with unclear cell outlines were observed. The β-galactosidase-positive cells in the aging model group were the blue-stained senescent cells. The cell morphology also changed significantly compared with the blank control group, which reduced the cell viability. In the experimental group, with the gradual increase in the SBP concentration, the cell morphology gradually returned to normal, the blue senescent cells were significantly reduced, and the number of abnormally shrunken cells within the visual field was also considerably reduced. Figure 2B shows that, compared with the control group, the cell viability of the H_2_O_2_-induced senescence model was significantly reduced. At the same time, the SBP experimental group could significantly improve the decreased cell viability induced by H_2_O_2_, and the effect was better at concentrations of 50 μg/mL and 100 μg/mL. This indicates that SBP could resist H_2_O_2_-induced cell senescence, mitigating the effects of aging on cell morphology and reducing the number of senescent cells.

### 3.3. Effects of SBP on ROS, SOD Activity, GSH, and MDA in H_2_O_2_-Induced Senescent HSFs

**Figure 3:**
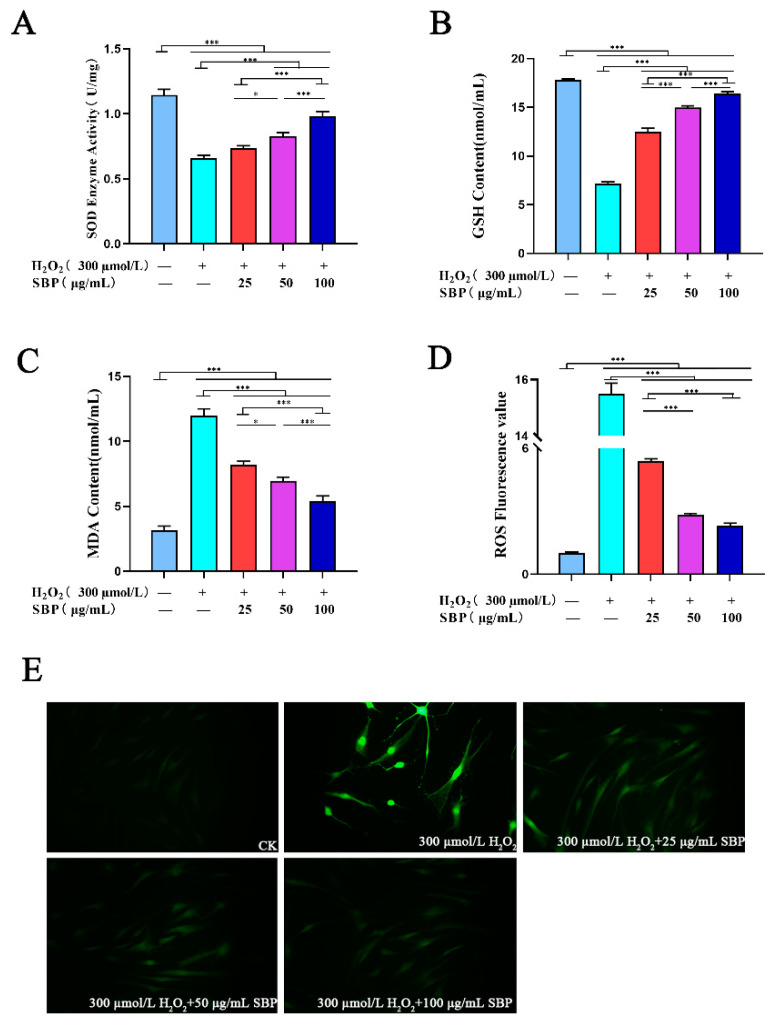
Effects of SBP on the SOD activity, GSH, MDA, and ROS content in H_2_O_2_-induced aging HSFs using related kits. (**A**–**D**): activity of SOD and contents of GSH, MDA, and ROS in cells treated with different experiments, respectively. (**E**) Fluorescence images of ROS in cells under different experimental conditions. In the figure “+” means added, “−” means not added. Results are the mean ± SD of three independent experiments. * *p* < 0.05, *** *p* < 0.001 indicating significant differences compared with the various experimental groups.

The intracellular oxidative stress response due to the increase in ROS is closely related to cellular senescence [19]. To explore the antioxidant effect of SBP in cell aging models, we detected the activity of SOD and the contents of GSH, MDA, and ROS in cells. As shown in Figure 3A–C, when compared with the blank control group, the SOD activity and GSH content in the cell senescence model were higher, while the MDA was much lower. SBP intervenes in the H_2_O_2_-induced senescence model by gradually increasing the SOD activity and GSH content with the increase in the SBP concentration. On the other hand, the MDA content showed a dose-dependent decrease. The ROS content was calculated by fluorescence spectrophotometry. Figure 3D,E shows that the H_2_O_2_-induced senescence model had the highest level of intracellular ROS and the brightest field of view. When compared with the H_2_O_2_-induced senescence model, the fluorescence intensity of the SBP experimental group and the blank sample group was darker, indicating that the intracellular ROS content was lower. These results suggest that the antioxidant properties of SBP can alleviate cellular senescence caused by H_2_O_2_-induced oxidative stress.

### 3.4. SBP Up-Regulates the Expression of Col I in H_2_O_2_-Induced Aging HSFs and Inhibits Col I Degradation by Regulating the Expressions of MMP-1, -3, and TIMP-1

**Figure 4:**
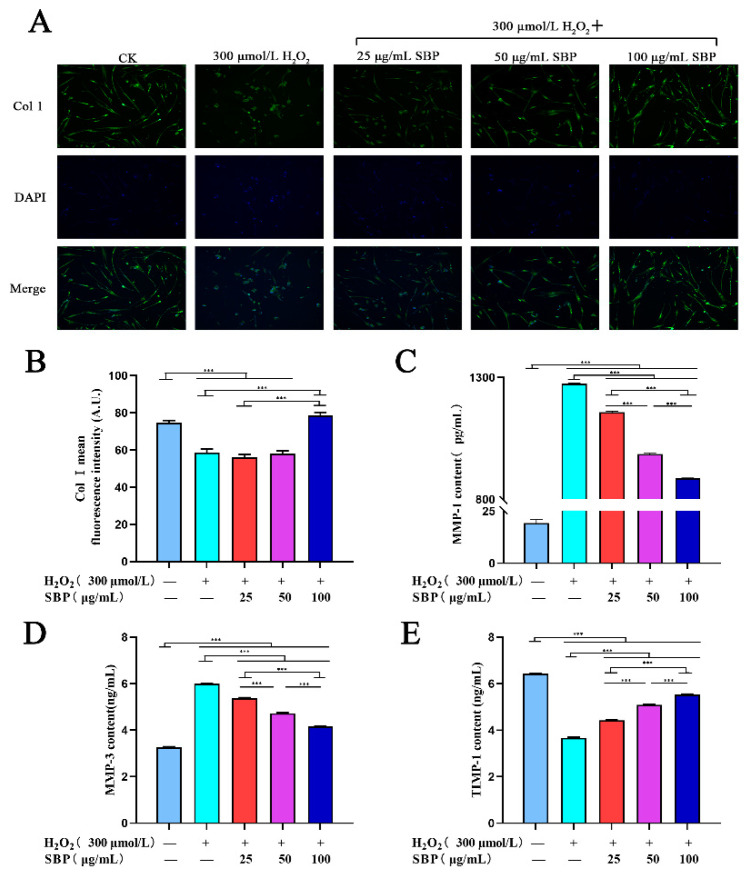
Effects of SBP on Col I expression in H_2_O_2_-induced senescent HSFs. (**A**): Representative immunofluorescence microscopy image showing the Col I levels in cells under different experimental treatments with Col I in green and DAPI-stained nuclei in blue. (**B**): Mean fluorescence intensity of Col I in cells under the different experimental conditions. (**C**–**E**): Concentrations of MMP-1, MMP-3, and TIMP-1 in different experimentally treated cells using the ELISA kit. In the figure “+” means added, “−” means not added. The results are the mean ± SD of three independent experiments. *** *p* < 0.001 indicate significant differences compared with the various experimental groups.

We verified that SBP positively affects Col I expression in H_2_O_2_-induced senescent HSFs by immunofluorescence staining. By combining Figure 4A,B, it can be concluded that Col I in the H_2_O_2_-induced senescence model had lower fluorescence intensity when compared with the blank control group. As for the experimental group, low to medium SBP concentrations (25 µg/mL and 50 µg/mL) did not affect the expression of Col I in senescent cells, while a high SBP concentration (100 μg/mL) could promote the expression of Col I in senescent cells. This indicated that a high concentration of SBP (100 µg/mL) could increase the expression of Col I in senescent cells.

The expression of MMP-1 and MMP-3 can affect the degradation of Col I, and their expression can be inhibited by TIMP-1. According to Figure 4C–E, the concentrations of MMP-1 and MMP-3 in the cellular aging model group increased significantly compared with the blank control group, while TIMP-1 decreased significantly. In the experimental groups, MMP-1 and MMP-3 decreased gradually with the gradual increase in the SBP concentration. On the contrary, the concentration of TIMP-1 in cells increased in a concentration-dependent manner with the gradual rise in the SBP concentration. Therefore, SBP can regulate the degradation of Col I through the expression of MMP-1, MMP-3, and TIMP-1 in cells.

### 3.5. SBP Promotes the Synthesis of Type I Procollagen through the TGF-β1/Smads Pathway

**Figure 5:**
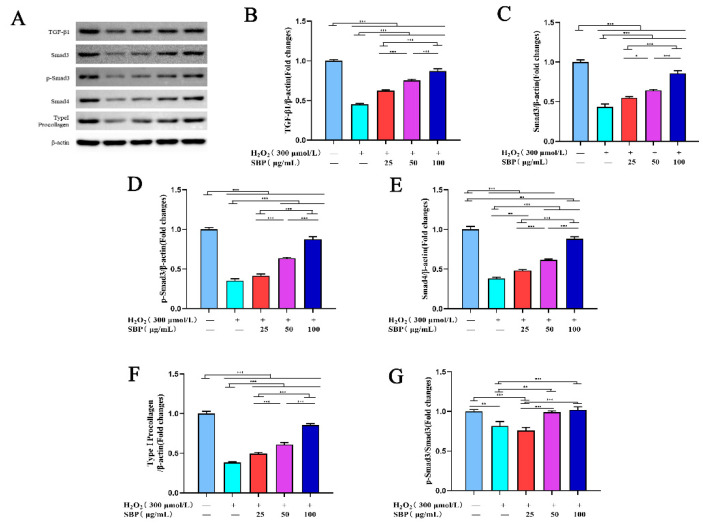
Effect of SBP on the synthesis of Type I procollagen by the TGF-β1/Smads pathway. (**A**): Protein bands of related proteins in the TGF-β1/Smads pathway. (**B**–**F**) TGF-β1, Smad3, p-Smad3, Smad4, and Type I procollagen, respectively. (**G**) shows the p-Smad3/Smad3 in cells under different experiments. In the figure “+” means added, “−” means not added. The results are the mean ± SD of three independent experiments. * *p* < 0.05, ** *p* < 0.01, and *** *p* < 0.001 indicate significant differences compared with the various experimental groups.

TGF-β1/Smads can regulate the synthesis of type I procollagen in cells, thereby affecting the synthesis and expression of Col I. Therefore, we investigated whether SBP stimulates the production of type I procollagen induced by TGF-β1 and its downstream effector Smads protein. As shown in Figure 5B–F, H_2_O_2_ inhibited the expressions of TGF-β1, Smad3, p-Smad3, Smad4, and type I procollagen and the ratio of p-Smad3/Smad3 in cells. However, as shown in the same figures, SBP reversed the effect of H_2_O_2_ on the above proteins. With the increase in the SBP concentration in the experimental group, the expression of the mentioned proteins in cells gradually increased. On the other hand, Figure 5G shows the inhibitory effect on the phosphorylation of Smad3. The low concentration of SBP (25 µg/mL) did not alleviate this inhibitory effect, while the medium and high concentrations (50 and 100 µg/mL) did(the higher the concentration of SBP, the better the effect). This result aligns with the conclusion of the previous part, proving that a high concentration of SBP (100 µg/mL) can increase the expression of Col I in senescent cells.

### 3.6. SBP Enhances the Migration Ability of H_2_O_2_-Induced Senescent HSFs

**Figure 6:**
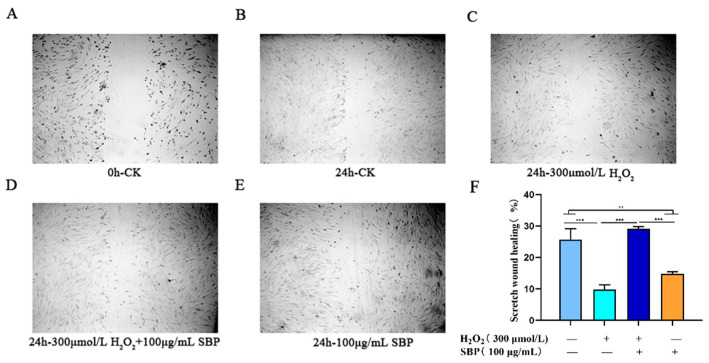
Effect of SBP on H_2_O_2_-induced senescent HSFs’ cell migration. (**A**): Microscopic observation of the blank control group at an incubation time of 0 h. (**B**): Microscopic observation of the blank control group at an incubation time of 24 h. (**C**): Microscopic observation of cells cultured for 24 h after adding 300 µmoL/L H_2_O_2_ to the culture medium. (**D**): Microscopic observation of cells cultured for 24 h after adding 300 µmoL/L H_2_O_2_ and 100 µg/mL to the culture medium. (**E**): Microscopic observation of cells cultured for 24 h after adding 100 µg/mL to the culture medium. (**F**): Cell migration rate of different experimental treatment groups after 24 h of cultivation. In the figure “+” means added, “−” means not added. The results are the mean ± SD of three independent experiments. ** *p* < 0.01, and *** *p* < 0.001 indicate significant differences compared with the various experimental groups.

This cell scratch experiment simulated the process of cell migration in vivo to a certain extent. By observing the peripheral cells’ growth (repair) to the central scratch area, we could determine the cells’ ability to grow, migrate, and repair. After 24 h, the cell migration rate was quantified. As shown in Figure 6 A–F, the migration rate of the H_2_O_2_-induced senescence model group was lower than that of the blank control group by 62%. The addition of 100 µg/mL SBP significantly improved the mobility of the aging model and approached the level of the blank control group. However, if 100 µg/mL SBP was added alone, the cell migration rate was about 43% lower than that of the blank control. This indicates that a high concentration of SBP (100 µg/mL) was beneficial to the growth and migration of senescent cells, but the effect was less significant on normal cells.

## 4. Discussion

Oxidative and inflammatory responses accompany the entire life process of the human body. When the accumulation of ROS exceeds the scavenging ability of the antioxidant defense system, peroxidative damage to tissue cells occurs [20]. The skin is part of the human body exposed to various external stimuli, such as smoke, alcohol consumption, ultraviolet radiation, and common pollutants. Excessive accumulation of ROS in cells leads to wrinkles, irregular hyperpigmentation, and accelerated skin aging [21]. Therefore, more and more research is focusing on the resistance effectiveness of natural substances toward oxidative stress damage to postpone aging. SBP is the most critical antioxidant component of *Sea buckthorn*, and our team’s previous research proved that SBP has an excellent free-radical-scavenging ability [15]. However, no studies have yet determined the ability of SBP to delay skin aging caused by oxidative stress. In order to further extend the use of SBP, the underlying mechanism of SBP’s anti-aging effect was explored. We used low, medium, and high concentrations (25, 50, 100 µg/mL) of SBP to treat senescent HSFs (induced by H_2_O_2_) and observed their effect on senescent HSFs, cell morphology, and cell viability with the help of the β-galactosidase staining method. SBP was found to reduce the number of senescent cells significantly. In addition, cell morphology was restored, and cell viability was increased with the help of SBP. To further verify the underlying mechanism, follow-up experiments were conducted.

We experimentally verified that SBP could achieve anti-aging effects by enhancing antioxidant enzyme activity and many non-enzymatic defense mechanisms to eliminate excess ROS generated by H_2_O_2_-induced cellular oxidative stress. SOD and GSH are critical body defense systems against oxidative stress. They are also crucial enzymes and antioxidants for scavenging ROS [22]. SOD is a strong superoxide anion (O^2–^) scavenger [23]. The unique structure of GSH makes it an important free radical scavenger in the body, and it plays a vital role in antioxidant, detoxification, immunity enhancement, and antitumor activity. Both of the above substances can protect the structure and function of the cell membrane from oxide damage and interference [24]. MDA is the product of lipid peroxidation. After excess ROS oxidizes saturated fatty acids in cells, they first form conjugated diene hydroperoxides (CD-POV), which are further oxidized to epoxides and then decomposed to MDA. Upon further oxidation, the concentration of MDA gradually increased [25]. The CD-POV, MDA, and oxidation product concentrations are often used as biomarkers of cellular oxidation [26]. Proanthocyanidins have a more powerful ROS scavenging capability than vitamin C, vitamin E, and beta-carotene, which can quickly neutralize ROS and reduce the damage to cells caused by excessively elevated ROS [27]. In our study, we found that H_2_O_2_ treatment increased the MDA content of HSFs and decreased the antioxidant capacity of SOD and GSH, resulting in excess reactive oxygen species in cells. After treatment with different concentrations of SBP, the up-regulation of the intracellular ROS level induced by H_2_O_2_ was significantly decreased, the contents of SOD and GSH increased, and the content of MDA decreased. These results suggest that SBP can enhance the activities of antioxidant enzymes and antioxidants, remove excess ROS effectively, reduce the oxidative stress-stimulated response in cells, and exert anti-aging effects.

Collagen is a ubiquitous natural substance in the human body that is responsible for maintaining much of the body’s structural integrity [28]. Col I is the main component secreted by fibroblasts into the extracellular matrix (ECM), which accounts for 70% of the dermis. It can maintain the elasticity and firmness of the skin [29,30], and acts as mechanical structural support for many human parts, such as bones, skin, tendons, ligaments, and blood vessels. It also facilitates the creation of a microenvironment for the development of immune responses in lymphoid organs. Moreover, it plays a vital role in regulating physiological processes, such as cell adhesion, proliferation, and differentiation [31]. One of the manifestations of human skin cell aging is the loss of Col I. Therefore, we verified the effect of SBP on the synthesis and degradation of Col I in H_2_O_2_-induced aging HSFs by two methods. On the one hand, we confirmed that SBP could facilitate the synthesis of Col I in H_2_O_2_-induced aging HSFs through the TGF-β1/Smads pathway. TGF-β1/Smads is a representative signaling pathway involved in collagen synthesis. TGF-β1 receptors can be divided into two types: TβRI and TβRII. When the ligand binds to the receptor, TβRI and TβRII combine to form a dimer. Then, TβRI is phosphorylated by TβRII. The activated TβRI recognizes and phosphorylates Smad3. Phosphorylated Smad3 forms a complex with Smad4 and then transfers to the nucleus to promote the synthesis of type I procollagen, which further promotes the synthesis of Col I [32]. The study by Cavinato and Jansen-Dürr et al. found that UVB irradiation increased the amount of ROS in cells, which in turn reduced TβRII to downregulate Smad3 phosphorylation and reduce collagen synthesis [33]. Therefore, we found that SBP restored the expression of TGF-β1 downregulated by the abnormal increase in ROS. SBP also stimulated the phosphorylation of Smad3, but we found that the effect of a low dose of SBP on the phosphorylation of Smad3 was not noticeable. Despite the less apparent effect of a low dose of SBP on Smad3 phosphorylation, the result is consistent with the quantitative determination of the Col I content in senescent HSFs by immunofluorescence staining. Therefore, we concluded that SBP positively affected the synthesis of Col I in senescent cells and was dose-dependent.

**Figure 7:**
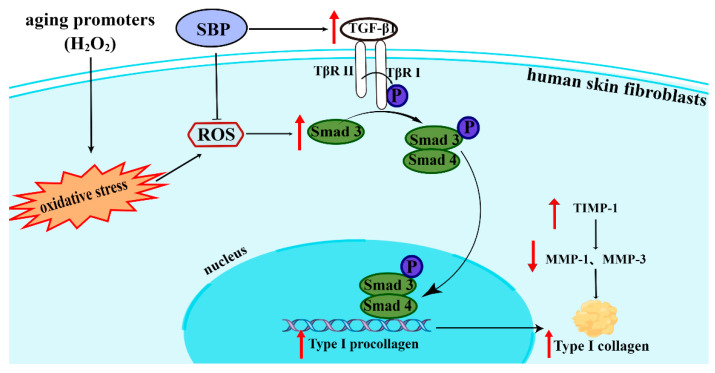
A possible mechanism by which SBP has anti-aging effects on the skin. SBP enhances Col I accumulation by modulating the TGF-β1/Smads pathway in HSFs and inhibits collagen degradation by regulating the levels of MMP-1, -3, and TIMP-1 (diagram was drawn using Figdraw).

On the other hand, we wondered if SBP affected the degradation of Col I. MMPs are a multigene family of metal-dependent endopeptidases that play important and diverse roles in normal physiological and pathological processes involving cell differentiation, proliferation, apoptosis, angiogenesis, and wound healing. Moreover, they can also modulate the activity of other proteases, growth factors, cytokines, and cell surface ligands and receptors [34]. The expression activity of MMPs is affected by the MAPK signaling pathway and inhibited by TIMP. The MAPK signaling pathway includes three critical proteins: ERK, JNK, and p38. Phosphorylated MAPK protein can upregulate the expression of c-Fos and c-Jun, and induce the activation of activator protein 1 (AP-1). The activated AP-1 acts as a nuclear transcription factor and binds to the promoter of MMPs to induce MMP gene transcription [35]. Heo and Lee et al. [36] found that the treatment of Hs68 cells with ginseng seed extract attenuated the UV-induced MMP-1 and MMP-3 increments through the MAPK signaling pathway to decrease collagen degradation. Since the primary function of MMPs is to degrade ECM, Col I degradation is initiated by MMP-1 and continued by MMP-3 [6]. The expression of MMP-1 and MMP-3 will be inhibited by TIMP-1 [7]. Therefore, the MMP/TIMP system is crucial in maintaining the ECM structure [37]. Thus, natural ingredients that can manipulate these biomarkers help improve cellular aging. In our study, we found that SBP could reduce the amounts of MMP-1 and MMP-3 by regulating the content of TIMP-1 in cells (caused by the excessive increase in ROS in cells), thereby inhibiting collagen degradation in senescent cells. In conclusion, our experiments found that SBP could upregulate collagen synthesis through the TGF-β1/Smads pathway and inhibit collagen degradation by regulating the MMP/TIMP system. Thus, it effectively prevents cellular senescence due to excessive ROS in cells (Figure 7).

## 5. Conclusions

We concluded that SBP isolated from *Sea buckthorn* seed meal exerted potent anti-aging activity in H_2_O_2_-induced aging HSFs. SBP could enhance the activities of antioxidant enzymes and antioxidants, remove excess ROS effectively, reduce the number of senescent cells, and significantly improve the changes in cell morphology and cell viability caused by excess ROS in skin cells. In addition, SBP could promote the synthesis of Col I in aging HSFs through the TGF-β1/Smads pathway and inhibit the degradation of Col I by regulating the MMPs/TIMPs system, thereby maintaining the stability of the ECM structure to achieve anti-aging purposes. Although 25, 50, and 100 µg/mL of SBP exhibited anti-aging properties, the highest concentration of SBP (100 µg/mL) exerted the best effects on senescent cells. Finally, we studied the migration ability of SBP, and the results showed that 100 µg/mL of SBP was more conducive to the cell migration of senescent cells, laying the foundation for our follow-up animal experiments. This study provides a deeper understanding of the mechanism behind SBP in delaying skin aging. These results will increase the application value of SBP in the cosmetic and antioxidant functional food industries.

## Data Availability

The original contributions presented in the study are included in the article; further inquiries can be directed to the corresponding author.

## References

[1] Park S. (2022). Biochemical, structural and physical changes in aging human skin, and their relationship. Biogerontology.

[2] Mo Q., Fu H., Zhao D., Zhang J., Wang C., Wang D., Li M. (2021). Protective Effects of Mogroside V on Oxidative Stress Induced by H_2_O_2_ in Skin Fibroblasts. Drug Des. Dev. Ther..

[3] Chung K.Y., Agarwal A., Uitto J., Mauviel A. (1996). An AP-1 binding sequence is essential for regulation of the human alpha2(I) collagen (COL1A2) promoter activity by transforming growth factor-beta. J. Biol. Chem..

[4] Ignotz R.A., Massagué J. (1986). Transforming growth factor-beta stimulates the expression of fibronectin and collagen and their incorporation into the extracellular matrix. J. Biol. Chem..

[5] Fisher G.J., Quan T., Purohit T., Shao Y., Cho M.K., He T., Varani J., Kang S., Voorhees J.J. (2009). Collagen fragmentation promotes oxidative stress and elevates matrix metalloproteinase-1 in fibroblasts in aged human skin. Am. J. Pathol..

[6] Sárdy M. (2009). Role of matrix metalloproteinases in skin ageing. Connect. Tissue Res..

[7] Escaff S., Fernández J.M., González L.O., Suárez A., González-Reyes S., González J.M., Vizoso F.J. (2010). Study of matrix metalloproteinases and their inhibitors in prostate cancer. Br. J. Cancer.

[8] Ciesarová Z., Murkovic M., Cejpek K., Kreps F., Tobolková B., Koplík R., Belajová E., Kukurová K., Daško Ľ., Panovská Z. (2020). Why is sea buckthorn (*Hippophae rhamnoides* L.) so exceptional? A review. Food Res. Int..

[9] Michel T., Destandau E., Floch G.L., Lucchesi M.E., Elfakir C. (2011). Antimicrobial, antioxidant and phytochemical investigations of sea buckthorn (*Hippophaë rhamnoides* L.) leaf, stem, root and seed. Food Chem..

[10] Wang T.K., Xu S., Li S., Zhang Y. (2020). Proanthocyanidins Should Be a Candidate in the Treatment of Cancer, Cardiovascular Diseases and Lipid Metabolic Disorder. Molecules.

[11] Wang J., Yu T., Sheng L., Zhang H., Chen F., Zhu J., Ding M. (2021). Lotus Seedpod Proanthocyanidins Protect Against Light-Induced Retinal Damage via Antioxidative Stress, Anti-Apoptosis, and Neuroprotective Effects. Med. Sci. Monit..

[12] Gong P., Wang P., Pi S., Guo Y., Pei S., Yang W., Chang X., Wang L., Chen F. (2022). Proanthocyanidins Protect Against Cadmium-Induced Diabetic Nephropathy Through p38 MAPK and Keap1/Nrf2 Signaling Pathways. Front. Pharm..

[13] Du H., Wang Q., Li T., Ren D., Yang X. (2021). Grape seed proanthocyanidins reduced the overweight of C57BL/6J mice through modulating adipose thermogenesis and gut microbiota. Food Funct..

[14] Fine A.M. (2000). Oligomeric proanthocyanidin complexes: History, structure, and phytopharmaceutical applications. Altern. Med. Rev..

[15] Zhu Y.L., Yuen M., Li W., Yuen H., Wang M., Smith D., Peng Q. (2021). Composition analysis and antioxidant activity evaluation of a high purity oligomeric procyanidin prepared from sea buckthorn by a green method. Curr. Res. Food Sci..

[16] Childs B.G., Bussian T.J., Baker D.J. (2019). Cellular Identification and Quantification of Senescence-Associated β-Galactosidase Activity In Vivo. Methods Mol. Biol..

[17] Menicacci B., Laurenzana A., Chillà A., Margheri F., Peppicelli S., Tanganelli E., Fibbi G., Giovannelli L., Del Rosso M., Mocali A. (2017). Chronic Resveratrol Treatment Inhibits MRC5 Fibroblast SASP-Related Protumoral Effects on Melanoma Cells. J. Gerontol. A Biol. Sci. Med. Sci..

[18] Pan C., Lang H., Zhang T., Wang R., Lin X., Shi P., Pang X. (2019). Conditioned medium derived from human amniotic stem cells delays H_2_O_2_-induced premature senescence in human dermal fibroblasts. Int. J. Mol. Med..

[19] Cui Y., Li F., Zhu X., Xu J., Muhammad A., Chen Y., Li D., Liu B., Wang C., Wang Z. (2022). Alfalfa saponins inhibit oxidative stress-induced cell apoptosis through the MAPK signaling pathway. Redox Rep: Commun. Free Radic. Res..

[20] Görlach A., Dimova E.Y., Petry A., Martínez-Ruiz A., Hernansanz-Agustín P., Rolo A.P., Palmeira C.M., Kietzmann T. (2015). Reactive oxygen species, nutrition, hypoxia and diseases: Problems solved?. Redox Biol..

[21] Blumberg J. (2004). Use of biomarkers of oxidative stress in research studies. J. Nutr..

[22] Forman H.J., Zhang H. (2021). Targeting oxidative stress in disease: Promise and limitations of antioxidant therapy. Nat. Rev. Drug Discov..

[23] Yan Z., Spaulding H.R. (2020). Extracellular superoxide dismutase, a molecular transducer of health benefits of exercise. Redox Biol..

[24] Li W., Li M., Qi J. (2021). Nano-Drug Design Based on the Physiological Properties of Glutathione. Molecules.

[25] Alyethodi R.R., Sirohi A.S., Karthik S., Tyagi S., Perumal P., Singh U., Sharma A., Kundu A. (2021). Role of seminal MDA, ROS, and antioxidants in cryopreservation and their kinetics under the influence of ejaculatory abstinence in bovine semen. Cryobiology.

[26] Wang H.R., Zhao X.Y., Zhang J.M., Lu C., Feng F.J. (2022). Arbuscular mycorrhizal fungus regulates cadmium accumulation, migration, transport, and tolerance in Medicago sativa. J. Hazard Mater..

[27] Huang X., Ye Y., Zhang J., Zhang X., Ma H., Zhang Y., Fu X., Tang J., Jiang N., Han Y. (2022). Reactive Oxygen Species Scavenging Functional Hydrogel Delivers Procyanidins for the Treatment of Traumatic Brain Injury in Mice. ACS Appl. Mater. Interfaces.

[28] Darvish D.M. (2022). Collagen fibril formation in vitro: From origin to opportunities. Mater. Today Bio.

[29] Zhang H., Shen F., Yu J., Ge J., Sun Y., Fu H., Cheng Y. (2022). Plasmodium vivax Protein PvTRAg23 Triggers Spleen Fibroblasts for Inflammatory Profile and Reduces Type I Collagen Secretion via NF-κBp65 Pathway. Front. Immunol..

[30] Shin J.W., Kwon S.H., Choi J.Y., Na J.I., Huh C.H., Choi H.R., Park K.C. (2019). Molecular Mechanisms of Dermal Aging and Antiaging Approaches. Int. J. Mol. Sci..

[31] Taemeh M.A., Shiravandi A., Korayem M.A., Daemi H. (2020). Fabrication challenges and trends in biomedical applications of alginate electrospun nanofibers. Carbohydr. Polym..

[32] Rittié L., Fisher G.J. (2002). UV-light-induced signal cascades and skin aging. Ageing Res. Rev..

[33] Cavinato M., Jansen-Dürr P. (2017). Molecular mechanisms of UVB-induced senescence of dermal fibroblasts and its relevance for photoaging of the human skin. Exp. Gerontol..

[34] Roy R., Morad G., Jedinak A., Moses M.A. (2020). Metalloproteinases and their roles in human cancer. Anat. Rec..

[35] Wen K.C., Fan P.C., Tsai S.Y., Shih I.C., Chiang H.M. (2012). Ixora parviflora Protects against UVB-Induced Photoaging by Inhibiting the Expression of MMPs, MAP Kinases, and COX-2 and by Promoting Type I Procollagen Synthesis. Evid. Based Complement. Altern. Med..

[36] Heo H., Lee H., Yang J., Sung J., Kim Y., Jeong H.S., Lee J. (2021). Protective Activity and Underlying Mechanism of Ginseng Seeds against UVB-Induced Damage in Human Fibroblasts. Antioxidants.

[37] Page-McCaw A., Ewald A.J., Werb Z. (2007). Matrix metalloproteinases and the regulation of tissue remodelling. Nat. Rev. Mol. Cell Biol..

